# Resistance of *Leishmania (Viannia) Panamensis* to Meglumine Antimoniate or Miltefosine Modulates Neutrophil Effector Functions

**DOI:** 10.3389/fimmu.2018.03040

**Published:** 2018-12-21

**Authors:** Ivo B. Regli, Olga Lucía Fernández, Berenice Martínez-Salazar, Maria Adelaida Gómez, Nancy Gore Saravia, Fabienne Tacchini-Cottier

**Affiliations:** ^1^Department of Biochemistry, WHO-Immunology Research and Training Center, University of Lausanne, Epalinges, Switzerland; ^2^Centro Internacional de Entrenamiento e Investigaciones Médicas, Cali, Colombia; ^3^CIDEIM, Universidad ICESI, Cali, Colombia

**Keywords:** *Leishmania*, neutrophils, drug resistance, miltefosine, antimony, NETs

## Abstract

*Leishmania (Viannia) panamensis (L. (V.) p.)* is the main causative agent of cutaneous leishmaniasis in Colombia and is usually treated with either meglumine antimoniate (MA) or miltefosine (MIL). In recent years, there has been increasing evidence of the emergence of drug-resistance against these compounds. Neutrophils are known to play an important role in immunity against *Leishmania*. These cells are rapidly recruited upon infection and are also present in chronic lesions. However, their involvement in the outcome of infection with drug-resistant *Leishmania* has not been examined. In this study, human and murine neutrophils were infected *in vitro* with MA or MIL drug-resistant *L. (V.) p*. lines derived from a parental *L. (V.) p*. drug-susceptible strain. Neutrophil effector functions were assessed analyzing the production of reactive oxygen species (ROS), the formation of neutrophil extracellular trap (NET) and the expression of cell surface activation markers. Parasite killing by neutrophils was assessed using *L. (V.) p*. transfected with a luciferase reporter. We show here that MA and MIL-resistant *L. (V.) p*. lines elicited significantly increased NET formation and MA-resistant *L. (V.) p*. induced significantly increased ROS production in both murine and human neutrophils, compared to infections with the parental MIL and MA susceptible strain. Furthermore, neutrophils exposed to drug-resistant lines showed increased activation, as revealed by decreased expression of CD62L and increased expression of CD66b in human neutrophils yet presented higher survival within neutrophils than the drug-susceptible strain. These results provide evidence that parasite drug-susceptibility may influences neutrophil activation and function as well as parasite survival within neutrophils. Further investigaton of the inter-relationship of drug susceptibility and neutrophil effector function should contribute to better understanding of the factors involved in susceptibility to anti-*Leishmania* drugs.

## Introduction

Cutaneous leishmaniasis is an important public health problem affecting 98 countries and territories worldwide. Approximately 350 million people live in endemic areas and over 2 million new cases are reported annually (1). In Colombia, *L. (V.) p*. is responsible for the majority of reported cases of cutaneous leishmaniasis (2) though *L. (V) braziliensis* may be more frequent among occupationally exposed military personnel (3). The first line of treatment in Latin America (4) and most of the world, consists of parenteral administration of meglumine antimoniate (MA) (5). Oral miltefosine (MIL) is generally administered as the second line treatment. In recent years, there has been an increasing number of reports of treatment failure and loss of susceptibility of clinical *Leishmania* strains to antimonial drugs, and association of resistance in some cases of therapeutic failure in leishmaniasis patients. The reported frequency of MA and MIL tolerance ranges from 10 to 70% and varies among *Leishmania* species and by geographic origin. The underlying mechanisms contributing to anti-leishmanial treatment failure in Colombia and the rest of the world are poorly understood (6–8).

It is well established that the outcome of anti-leishmanial treatment is not solely dependent on parasite drug-susceptibility, but also on intrinsic host factors. The interplay between parasites, the host and the immune response is also a factor determining the clinical outcome of *Leishmania* infection and treatment (9, 10). There is currently a lack of information on the role of neutrophils in *Leishmania* infection and its impact on drug-susceptibility. Recently, high levels of transcriptional signatures associated with chemokines promoting neutrophil recruitment were observed in *L. (V.) p*. infected primary macrophages and in chronic cutaneous lesions of CL patients, suggesting a potential role for neutrophils in the chronicity of infection (11).

Neutrophils are essential phagocytes of the innate immune system and the most abundant leukocytes in human circulation (12). They usually are the first cells recruited to the site of infection upon pathogen entry into a host (13). Neutrophils possess three major effector killing strategies: phagocytosis and subsequent production of reactive oxygen species (ROS), the release of granules that contain microbicidal proteins (14) and the formation of neutrophil extracellular traps (NETs). NETs are fibrous structures composed of dsDNA coated with a variety of different microbicidial proteins that can trap and kill a variety of pathogens (15). Upon phagocytosis of pathogens, neutrophils produce different reactive oxygen species that are synthesized by the enzymatic activity of NADPH-synthase (16). Indeed, a lack of NADPH-function manifests itself in higher susceptibility to a variety of infections and is known as chronic granulomatous disease (17). Also a lack of Myeloperoxidase (MPO), an enzyme that catalyzes the synthesis of highly microbicidal hypochlorous acid, is thought to lead to increased susceptibility to infection (18). Neutrophil granules can either fuse with pathogen containing phagosomes or be released into the extracellular space (19).

Increasing evidence points toward a crucial role of neutrophils in leishmaniasis (20, 21). It has been shown that neutrophils are massively recruited to the site of infection upon infection with *L. major* (22–25), *L. amazonensis* (26), *L. brazilensis* (27), *L. mexicana* (28), *L. infantum* (29), and *L. donovani* (30, 31) and that a subset of *L. mexicana* can even use these cells to replicate (32). While neutrophils are well known to have a protective role in many infections they can have either beneficial or detrimental roles in leishmaniasis depending on the *Leishmania* spp. involved (21). In most studies performed in murine models, the lack of neutrophils was beneficial to disease outcome in *Leishmania* infection. Furthermore, parasites are known to use these cells as “Trojan horses” to enter silently into the host macrophages (33). In contrast, neutrophil presence has been reported to positively affect disease outcome in the case of *L. braziliensis* (25, 34–36), *L. amazonensis* (37) and *L. donovani* infection (38).

Here, using both human and mouse neutrophils, we have investigated *in vitro* the role of these cells in the context of anti-*Leishmania* drug resistance following *L. (V.) p* infection, focusing on whether drug resistance could be linked to elicitation of distinct neutrophil activation profiles.

We found that *L. (V.) p*. lines with divergent drug-susceptibility to miltefosine and meglumine antimoniate have different effects on neutrophil phenotype and functionality. Our data show that neutrophils are differentially activated by drug-resistant and drug-susceptible parasites and that drug-resistant parasites are less affected by neutrophil microbial function than drug-susceptible parasites. These differences in the elicitation of neutrophil function and parasite killing may influence anti-leishmanial drug therapy.

## Materials and Methods

### Mice

C57BL/6 mice (5–10 weeks old) were purchased from Envigo (Cambridgeshire, United Kingdom) and bred under specified pathogen-free conditions at the animal facility of the University of Lausanne in Epalinges.

### Ethics Statement

All procedures involving human blood samples were approved by the Ethical Committee of the Canton of Vaud (CER-VD 2017-00182). Written informed consent was obtained from the healthy blood donors participating in this study. The study was conducted in compliance with the legislation of the Canton of Vaud and the Swiss Confederation as well as the declaration of Helsinki. Animal experimentation protocols were approved by the veterinary office of the Canton of Vaud (Authorization 1266.6-7 to F.T-C.) and were done in accordance to cantonal and federal law as well as the principles of the declaration of Basel. This study and the use of patient derived *Leishmania* strains and lines derived from these strains were approved by the Ethics Committees of CIDEIM- Colombia, in accordance with the national and international guidelines for Good Clinical Practice.

### Leishmania (Viannia) Panamensis Parasites

The *L. (V.) p*. strain (MHOM/COL/86/1166LUC) WT-control was used to derive the strains resistant to potassium antimony (III) tartrate hydrate (39) and miltefosine as described previously (40). Briefly, *L. (V.) p*. promastigotes were cultured in media containing increasing concentrations of drugs, either up to 2885 μM Sb or up to 60 μM of miltefosine. Upon establishment of drug-resistance, the strains were cultured in RPMI (Gibco) with 10% FCS and 1% PNS at 26°C. Antimony resistant parasites (MHOM/COL/86/ LUC 1166-1000.1) were cultured in medium containing 1 mM/L Sb^V^. The miltefosine resistant parasites (MHOM/COL/86/1166-LUC056) were cultured in medium containing 60 μM/L miltefosine. Luciferase expressing strains were grown in medium containing 60 μM G418 (Sigma).

### Snarf-1 Staining of Leishmania (Viannia) Panamensis Parasites

Parasites were stained with the intracellular dye SNARF-1 (Thermofisher) to render them fluorescent. Parasites were incubated in 6 μM solution of SNARF-1 in PBS for 30 min and subsequently washed twice. SNARF-1 stained fluorescent parasites were detected at an excitation wavelength of 488 nm and an emission wavelength of 610 nm.

### Drug-Susceptibility Screening

Drug-susceptibility of parasites was measured as a reduction of intracellular *L. (V.) p*. amastigote burden in macrophages derived from a U-937 cell line as described previously (40). Briefly, 1.2 × 10^5^ U-937 cells were treated with phorbol 12-myristate 13-acetate (PMA; 100 ng/ml; Sigma) to differentiate them into macrophages. Cells were co-cultured with *L. (V.) p*. promastigotes opsonized with 10% AB positive human serum at a MOI of 5:1 and incubated for 24 h at 34°C. Subsequently, the culture medium was replaced with RPMI containing either 16 μM miltefosine or 32 μg/ml Sb(V) and the cells were incubated for another 48 h. The Sb-containing medium was replaced after 24 h whereas the miltefosine-containing medium was not replaced during the 48 h of incubation. Parasite burden was assessed by light microscopy in four replicates by 2 microscopists in a blinded manner. The cut-offs defining sensitive respectively resistant strains were based on a previously published analysis (40). A dose response survival curve was done to establish the half maximal response of the drugs on intracellular amastigote macrophage killing (EC50), as previoulsy described (8). Briefly, the EC50 in presence of miltefosine for the WT *L. (V.) p*. strain was 2.1 μM/liter and the macrophage infection was 67% for the *L. (V.) p* MIL-R stain; the EC50 was >32 μM/liter with 70% of macrophage infection. In presence of Sb(V), the EC50 for the WT *L. (V.) p*. was 10 μM/liter with 73% macrophage infection, and for the *L. (V.) p*. MA-R strain the EC50 was >128 μM/liter with macrophage infection of 69% (mean of three independent experiments).

### Isolation Metacyclic *Leishmania (Viannia) Panamensis* Parasites

Metacyclic *L. (V.) p*. parasites were isolated using a Percoll (GE Healthcare) density gradient. Parasites were resuspended in 45% Percoll-medium suspension and layered onto 60% and 90% Percoll-medium suspension phases. After centrifugation without brakes at 4300 g for 45 min, metacyclic parasites were isolated from the 45%/60% Percoll-medium suspension interphase (41). The parasites were washed twice and counted using a Neubauer chamber.

### Human Neutrophil Isolation

Peripheral blood neutrophils were isolated from venous blood of healthy volunteers. Density gradient centrifugation using polymorphprep (Progen) was performed and the enriched neutrophils were isolated according to the manufacturer's instructions. The remaining red blood cells were lysed using ACK-Buffer. Neutrophil purity was assessed using Cyto-Spin and Quick-Fix staining and was established to be ≥95%. Twenty-four hours post culture, 70% of neutrophils were viable (DAPI-). Infection with parasites, independently of the susceptibility of the parasites diminished neutrophil viability to 50–60%.

### Murine Neutrophil Isolation

Bone marrow of the femora and tibia of C57BL/6 mice were flushed with RPMI. Erythrocytes were lysed with ACK buffer, the remaining leukocytes were washed and the neutrophils were then isolated by negative MACS using the neutrophil isolation kit (Miltenyi Biotec) according to the manufacturer's indications. At 24 h of culture, 90% of neutrophils were viable (DAPI^−^).

### Assessment of Net Formation

3 × 10^5^ Neutrophils were seeded on poly-L-Lysine coated coverslips, primed with 25 ng/mL GM-CSF and exposed for 4 h with either *L. (V.) p.*, PMA (Phorbol-12-myristate-13-acetate, Sigma), PMA + DNase (Sigma) or without a stimulus. Subsequently, cells were fixed with 4% PFA (Paraformaldehyde, Sigma) and stained with rabbit anti-human MPO (Dako) primary antibody and Alexa Fluor-488 goat anti-rabbit secondary antibody (Life Technologies). Coverslips were mounted on glass slides using a DAPI containing mounting medium (Molecular Probes) and analyzed by confocal microscopy (ZEISS LSM 510). To quantify NET formation, NETs were counted using fluorescence microscopy in a blinded manner based on at least in three replicates. NET formation was also assessed through the measurement of dsDNA in the supernatant using the Quant-iT PicoGreen kit (Thermo Fisher) by adapting a technique previously described (42). 2 × 10^6^ neutrophils were incubated for 4 h in X-vivo medium (Lonza) with either *L. (V.) p*., PMA or PMA + DNase or without stimulus. After incubation, the cell suspensions were centrifuged, and the supernatants were collected and transferred into black 96-well plates (Perkin Elmer). The picogreen dye was added and fluorescence was measured in using a plate reader (Molecular Devices, SpectraMax MiniMax 300) at an excitation wavelength of 480 nm and an emission wavelength of 520 nm.

### Measurement of Reactive Oxygen Species Production

ROS production was measured using a luminol-based chemiluminescence assay. 5 × 10^5^ neutrophils were incubated with either *L. (V.) p*., PMA or without stimulus in white, opaque 96-well plates (Perkin Elmer). Luminol (Carbosynth) was added at a final concentration of 20 μg/mL. ROS induced chemiluminescence was measured at all wavelengths with a plate reader at 5 min intervals during 1 h.

### Parasite Viability Measurement With Luciferase Assay

5 × 10^5^ neutrophils were infected with luciferase-expressing *L. (V.) p*. strains with different drug-susceptibility phenotypes during 2 h at 34°C at a MOI of 5:1, washed and subsequently incubated for 24 h. Parasite viability after incubation was measured using a luciferase assay system (Promega) according to the manufacturer's instructions. To measure parasite viability in presence or absence of NETs, neutrophils were incubated with or without 10 μg/ml DNase and parasite survival measured by chemi-luminescence as previously described (28). Chemiluminescence was measured in white, opaque 96-well plates in a plate reader (Molecular Devices, SpectraMax MiniMax 300) at all wavelengths.

### Identification of Neutrophil Cell Surface Markers and Measurement of Cell Viability by Flow Cytometry

Surface activation markers and cell viability markers in blood derived human neutrophils were analyzed using Flow Cytometry analyzer of either the BD LSR II or the BD LSR Fortessa series (Becton Dickinson) and analyzed with FlowJo software (Tree Star). The following antibodies were used: Anti-human: CD15-APC, CD62L-PerCP-efluor710, CD66b-PE-Cy7. Anti-mouse: Ly6G(1A8)-APC-Cy7, CD62L-PE, CD11b-PE-Cy7 (all from e-Bioscience). Cell viability: Live/Dead fixable Aqua Dead Cell Stain Kit (Invitrogen).

## Results

### Increased Reactive Oxygen Species Production by Murine and Human Neutrophils Challenged With Miltefosine-Resistant *Leishmania (Viannia) Panamensis* Strains

**Figure 1:**
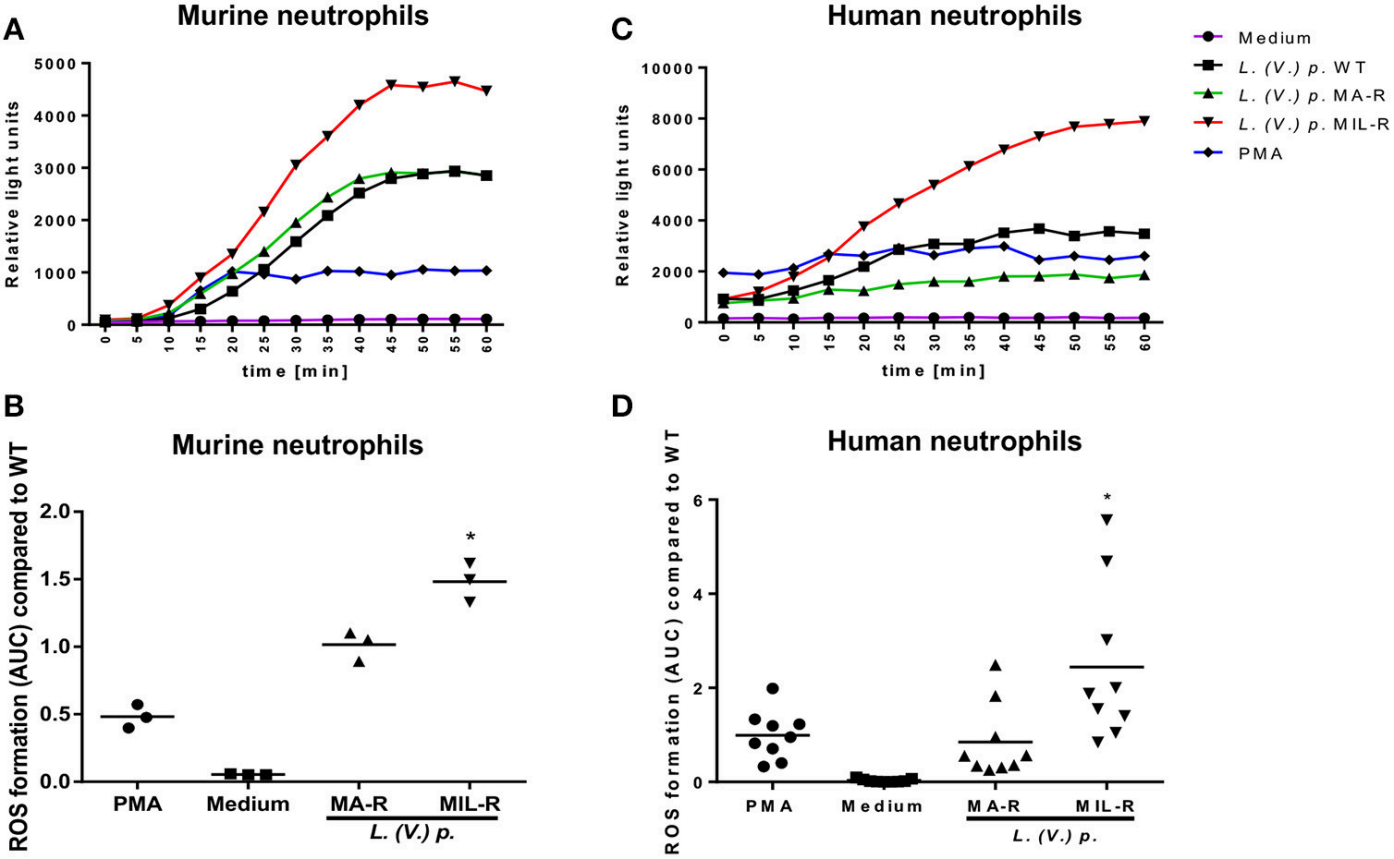
Miltefosine (MIL)-resistant *L. (V.) p*. parasites induce more ROS production in murine and human neutrophils than the meglumine antimony (MA)-resistant or drug susceptible strains. ROS production was measured using a luminol based chemiluminescence assay. Bone marrow derived murine neutrophils and blood-derived human neutrophils from healthy donors were incubated with either MIL-, MA-resistant or susceptible *L. (V.) p*., PMA or without stimulus. Luminol was added and ROS-induced chemiluminescence was measured with a plate reader every 5 min during 1 h. **(A,C)** One representative curve of ROS-induced luminol chemiluminescence values in murine **(A)** and human **(C)** neutrophils. **(B,D)** The corresponding area under the curve (AUC) at 60 min of measurement for murine **(B)** and human **(D)** neutrophils. ^*^p < 0.05, ^*^a MIL-R compared to WT, ^*^b MIL-R compared to MA-R, WT, wild type; MA-R, Resistant to meglumine antimoniate; MIL-R, Resistant to miltefosine; the data are pooled from three (human) or two (mouse) independent experiments.

To investigate the contribution of neutrophils in the intracellular survival of *Leishmania* during drug exposure, we first investigated whether *L. (V.) p*. strains susceptible or resistant to MA or MIL would induce different phenotype and function in neutrophils. The production of ROS by neutrophils is known to be an important defense mechanism against pathogens (43) and also contributes to NET formation (44). Therefore, we analyzed ROS production during the first hour of incubation of neutrophils with the different *L. (V.) p*. populations. Parasites that are resistant to miltefosine induced 1.5 times more ROS production compared to drug-susceptible parasites in BM-derived murine neutrophils (Figures 1A,B) and more than two times as much ROS production than drug-susceptible parasites in blood-derived human neutrophils from healthy donors (Figures 1C,D). In contrast, the induction of ROS by antimony resistant parasites strains in neutrophils did not differ from drug-susceptible *L. (V.) p*. parasites. Even though all *L. (V.) p*. strains induced ROS in neutrophils, these results show that the drug-susceptibility phenotype, specifically MIL-resistance, influences neutrophil ROS production.

### Drug-Resistant *Leishmania (Viannia) Panamensis* Induce More Neutrophil Extracellular Trap Formation Than Drug-Susceptible Strains in Murine and Human Neutrophils

**Figure 2:**
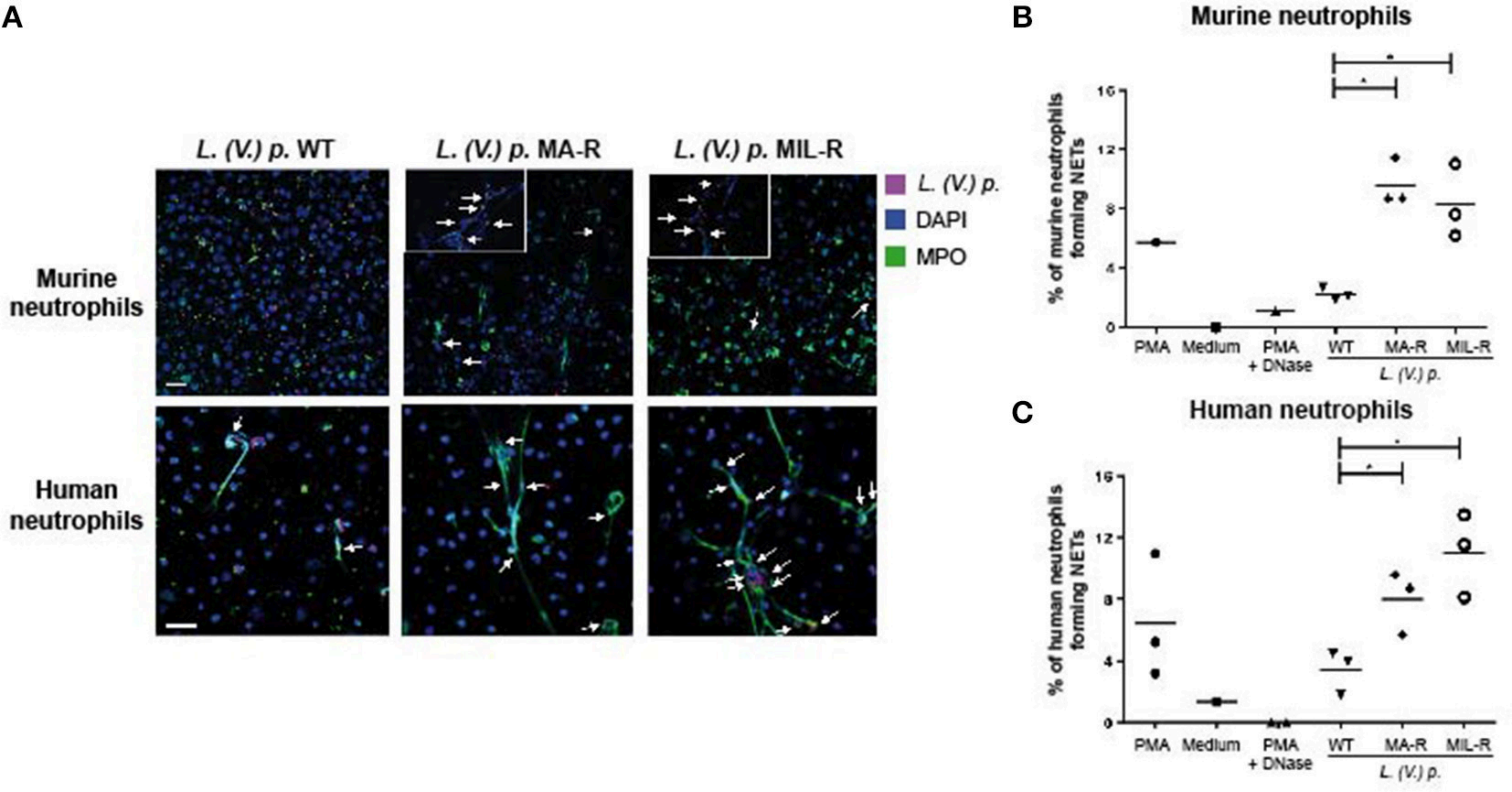
Murine and human neutrophils form more NETs when exposed to *L. (V) p*. strains that are resistant to either meglumine antimonate or miltefosine. Neutrophils were seeded on poly-L-Lysine coated coverslips and exposed for 4 h with either *L. (V.) p*.of the indicated drug suceptibility, PMA, PMA + DNase or without stimulus. Subsequently, cells were fixed, stained with anti-human MPO and DAPI **(A)** Confocal microscopy images of neutrophils upon exposure to *L. (V.) p*. murine or human neutrophils exposed to strains of SNARF-1 labeled *L. (V.) p*. promastigotes lines that were either resistant or susceptible to meglumine antimonate (MA) or miltefosine (MIL) at a 5:1 parasite-cell MOI. **(B,C)** The frequency of murine **(B)** or human **(C)** neutrophils forming NETs was counted with a fluorescence microscope. ^*^p < 0.05, each point corresponds to one healthy blood donor or one mouse. One representative experiment out of four is shown. The arrows point to colocalization of parasites with NETs. Zoom in of NETs are shown for murine neutrophils -The scale bar corresponds to 50 μM.

**Figure 3:**
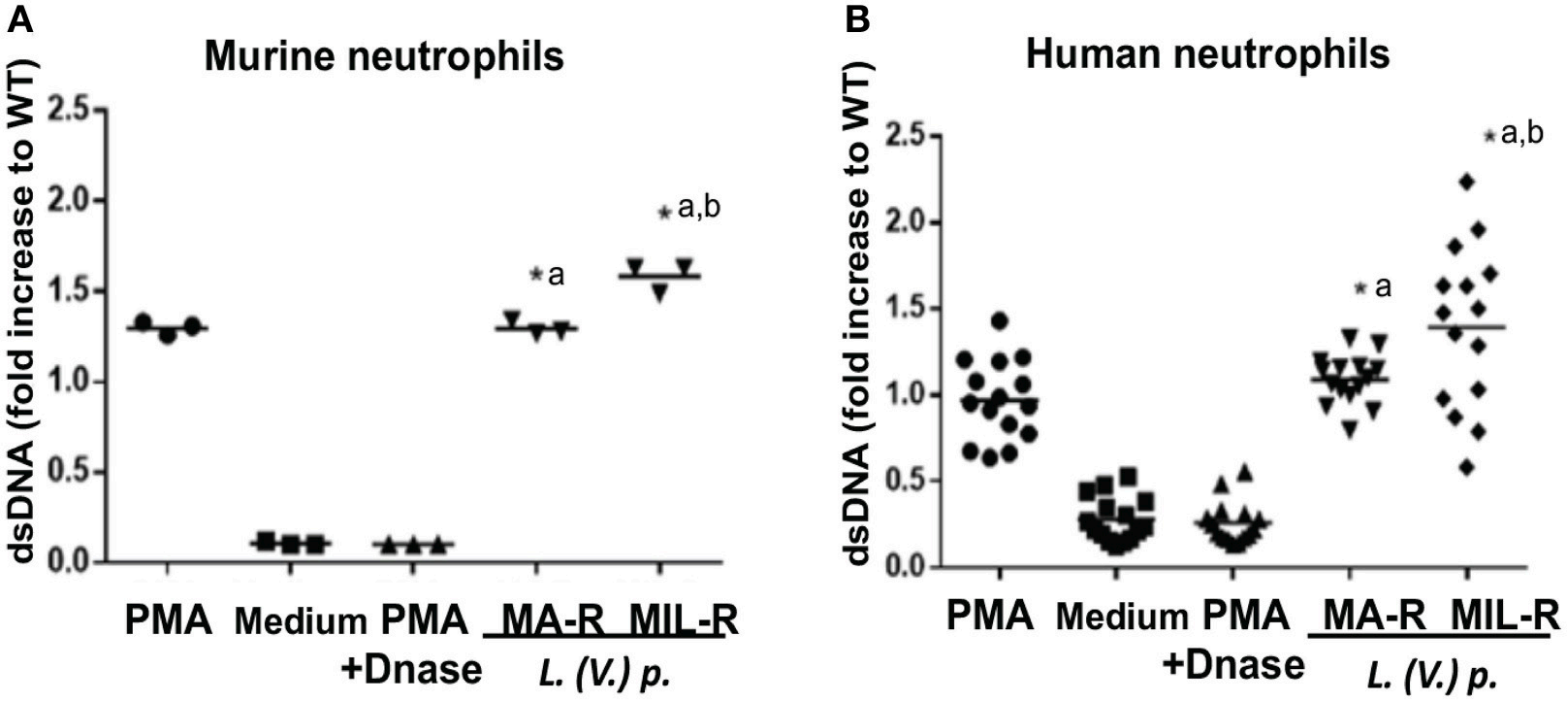
Murine and human neutrophils release more dsDNA when exposed to *L. (V) p*. strains that are resistant to either MA or MIL. Bone marrow derived murine neutrophils **(A)** and blood-derived human neutrophils from healthy donors **(B)** were exposed to *L. (V.) p*. of the indicated susceptibility at a 5:1 MOI. As controls, incubations with PMA, PMA + DNase or without stimulus were also performed. Four hours later, NET formation was quantified by measuring the levels of double stranded DNA (dsDNA) released in the supernatant using the PicoGreen fluorescent dye assay. ^*^p < 0.05, ^*^a MA-R or MIL-R compared to WT, ^*^b MIL-R compared to MA-R, Each point corresponds to one healthy blood donor or one mouse. One representative experiment out of four (human) and three (mice)independent experiments is shown.

Next, to visualize NETs, neutrophils were infected with *L. (V.) p*. of different drug susceptibility and NET formation analyzed by confocal microscopy. NETs were stained with DAPI to detect DNA filaments and an MPO mAb to detect the MPO associated with it. All parasite populations tested induced NET formation in both murine and human neutrophils as observed by DNA-MPO co-localization (Figure 2A). Drug resistant parasites induced substantially more NETs (two to three times) in murine and human neutrophils compared to drug-susceptible parasites (Figures 2B,C). We observed parasites in association with the NETs, a process more easily detectable in human NETs. To confirm these results, we used the picogreen assay that measures the amount of dsDNA released by neutrophils into the supernatant (42). We again observed that drug-resistant *L. (V.) p*. parasites induced a significantly higher amount of dsDNA release compared to drug-susceptible parasites in human and murine neutrophils (Figure 3). Collectively, these results demonstrate that MIL and MA-resistant *L. (V.) p* lines induce more NET formation than the parental drug-susceptible strain.

### Modulation of Neutrophil Activation Markers by *Leishmania (Viannia) Panamensis* of Different Drug-Susceptibility Phenotypes

**Figure 4:**
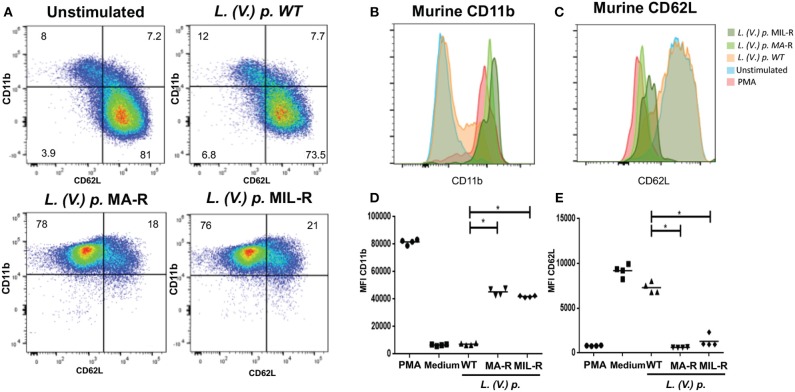
Expression of cell surface activation markers of murine neutrophils in response to exposure to *L. (V.) p*. promastigotes with different drug-susceptibility phenotypes. Bone marrow derived murine neutrophils were exposed to the indicated *L. (V.) p*. lines at a MOI of 1:10 or without stimulus for 90 min. Incubation with PMA was used as a positive control. Neutrophils were then stained with the CD11b and CD62L mAbs and surface expression was analyzed by flow cytometry. **(A)** Representative flow cytometry plot indicating CD11b and CD62L positive and negative populations. **(B,C)** A representative histogram plot of normalized fluorescence values of murine neutrophils stained with antibodies against CD11b and CD62L is shown. Normalized fluorescence values of murine neutrophils stained with antibodies against CD11b and CD62L is shown. **(C,D)** MFI values of murine neutrophils stained with antibodies against CD62L and CD11b. WT, Wild type; MA-R, Resistant to meglumine antimoniate; MIL-R, Resistant to miltefosine. The data is representative of three experiments. ^*^p < 0.05.

**Figure 5:**
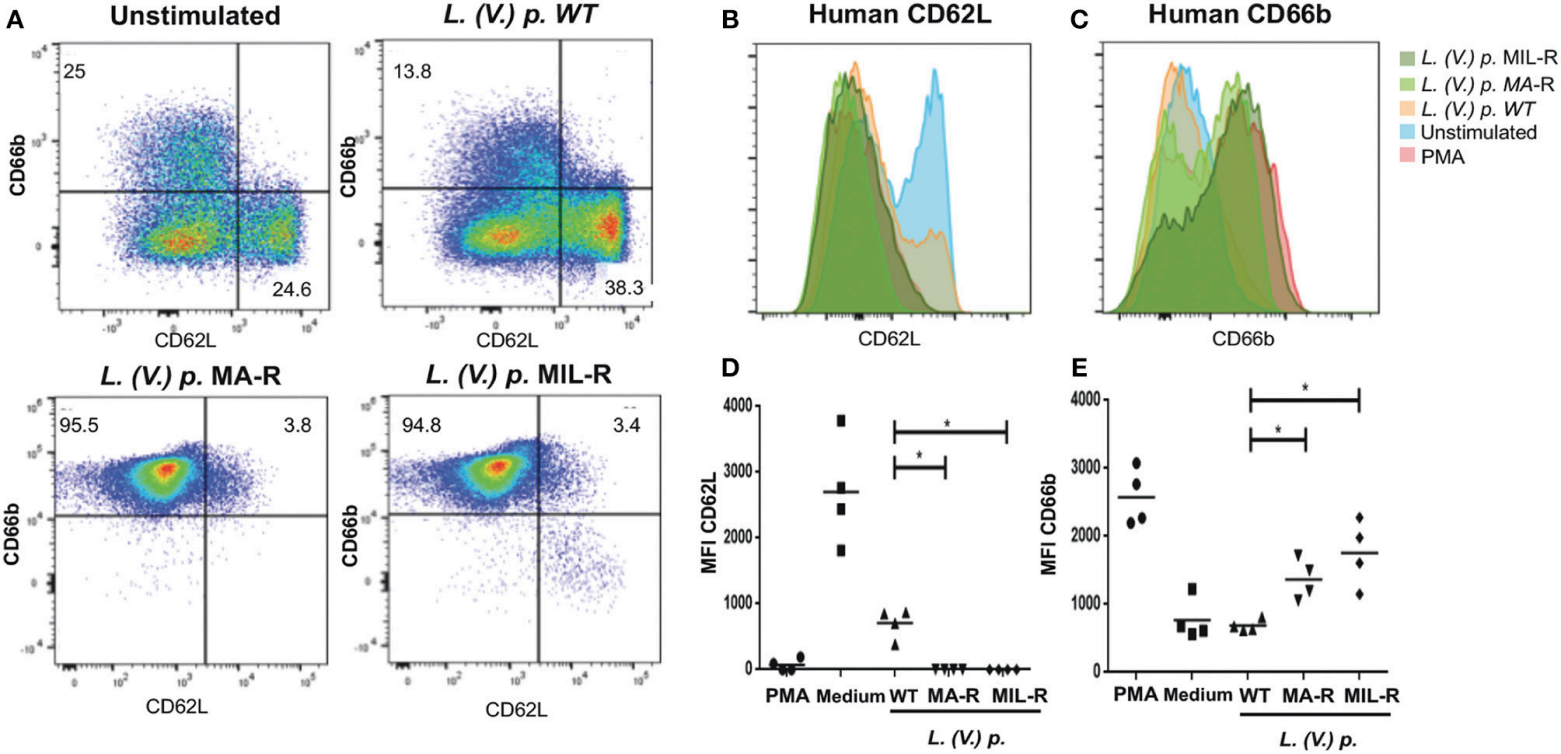
Expression of surface activation markers of human neutrophils in response to exposure to *L. (V.) p*. metacyclic promastigotes of different drug-susceptibility phenotypes. Blood-derived neutrophils from healthy donors were exposed to the indicated *L. (V.) p*. lines at a MOI of 1:10 or without stimulus for 90 min. Incubation with PMA was used as a positive control. Neutrophils were then stained with the CD62L and CD66b mAbs and surface expression was analyzed by flow cytometry. **(A)** Representative flow cytometry plot indicating CD62L and CD66b positive and negative populations. **(B,C)** One representative flow cytometry plot of normalized fluorescence values of human neutrophils stained with antibodies against CD62L and CD66b that were exposed to different *L. (V.) p*. strains at a MOI of 10. Is shown **(D,E)** MFI values of human neutrophils stained with antibodies against CD62L and CD11b. WT, Wild type; MA-R, Resistant to meglumine antimoniate; MIL-R, Resistant to miltefosine. The data is representative of a total of five experiments. ^*^p < 0.05.

We next investigated if exposure of neutrophils to *L. (V.) p*. of different drug-susceptibility would result in distinct modulations of cell surface markers. Using flow cytometry, we observed that BM-derived murine neutrophils exposed to MIL and MA drug-resistant *L. (V.) p* lines expressed more CD11b (Mac-1) and CD62L (L-selectin) on their surface than neutrophils exposed to drug-susceptible *L. (V.) p*. (Figure 4). CD62L and CD66b (exocytosis of secondary granules) expression by human neutrophils derived from the blood of healthy individuals exposed to drug-susceptible and resistant *L. (V.) p* was analyzed by flow cytometry (Figures 5A–C). MA and MIL resistant parasites elicited a decrease in expression of CD62L and an increase in expression of CD66b (Figures 5D–E). These data show that both human and murine neutrophils exposed to drug-resistant *L. (V.) p*. lines are activated to a greater extent and have a phenotype that is more associated with extravasation, compared to neutrophils challenged with drug-susceptible *L. (V.) p*.

### Drug-Resistant *Leishmania (Viannia) Panamensis* Strains Are More Resistant to Neutrophil Parasite Killing 24 h After Infection

**Figure 6:**
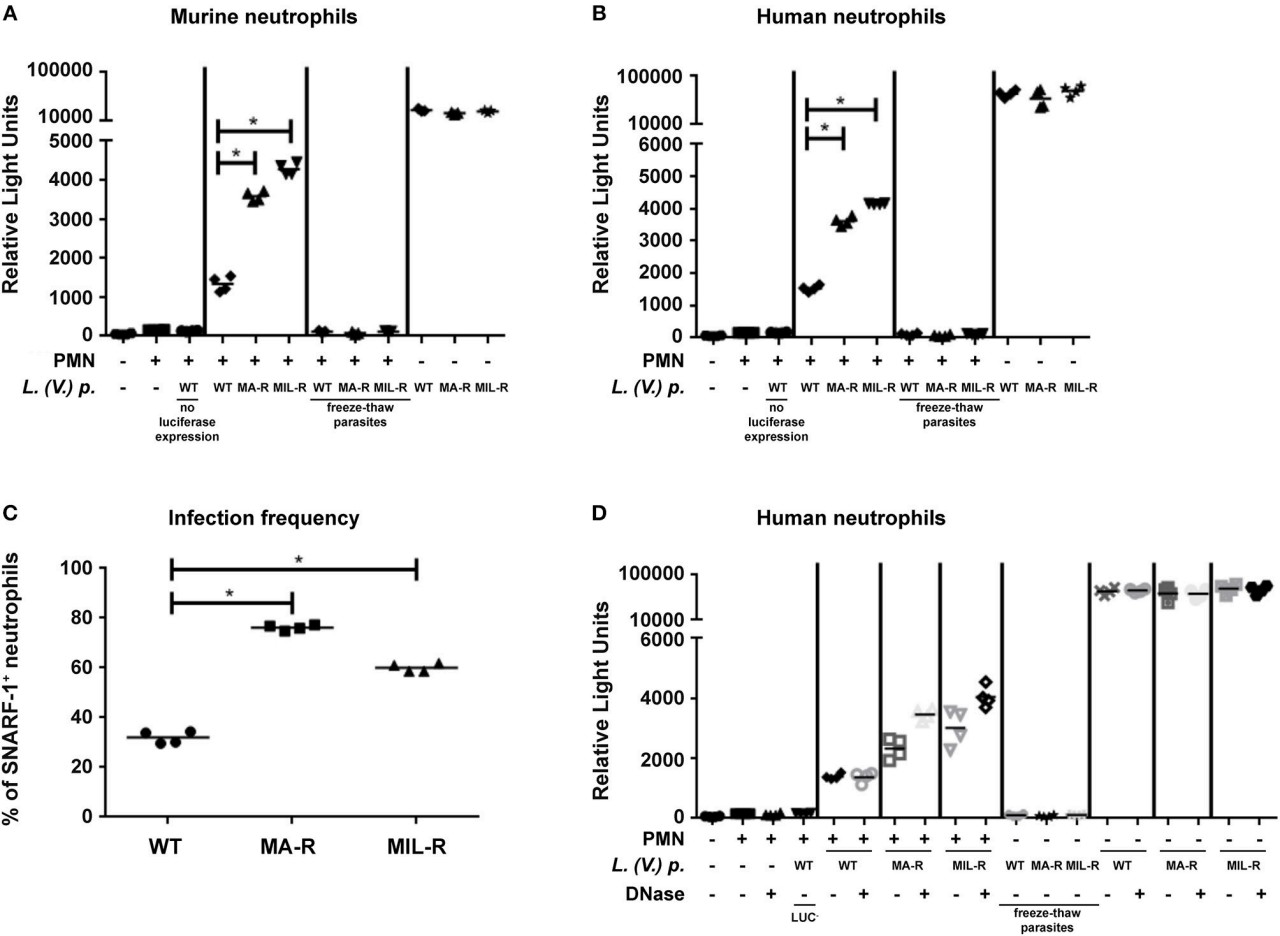
Killing of *L. (V.) p*. of different drug susceptibilities by murine and human neutrophils. Neutrophils were infected with luciferase expressing *L. (V.) p*. lines with different drug-susceptibility phenotypes for 2 h at 34°C, washed and further incubated for 24 h prior to analysis. Parasite viability after incubation was measured using a luciferase assay system. Murine **(A)** and human **(B)** neutrophils were exposed to luciferase expressing *L. (V.) p*. of the indicated drug susceptibility at a 5:1 MOI. 24 h later chemiluminescence was measured represented as relative light units and parasite killing was assessed MIL-R: Resistant to miltefosine, MA-R: Resistant to meglumine antimoniate, freeze-thaw parasites were killed by 10 times of subsequent freezing and thawing, PMN: Neutrophils. One representative experiment is shown out of four. **(C)** Infection frequency of neutrophils 4 h after exposure to the parasites of the indicated drug susceptibility. **(D)** Assessment of parasite killing by NETs. Human neutrophils were exposed to parasites of the indicated susceptibility as in **(B)**, but in addition, DNAse was added to the neutrophils as indicated. This are representative experiments of two.

As we observed differences in ROS production, NET formation and neutrophil activation in neutrophils exposed to *L. (V.) p*. of different drug-susceptibility phenotypes, we assessed whether this would have an impact on parasite killing. Luciferase expressing *L. (V.) p*. lines of the indicated drug-susceptibility phenotypes were exposed to murine or human neutrophils for 24 h and parasite viability was analyzed by measuring luciferase activity. Drug-resistant parasites were significantly more resistant to neutrophil killing by murine and human neutrophils than the drug-susceptible strain (Figures 6A,B). The frequency of neutrophil infection observed 4 h after infection with MA-R and MIL-R *L. (V.)* was significantly higher for drug-resistant parasites than WT parasites (Figure 6C) and this difference persisted 24 h post infection, suggesting diminished susceptibility to neutrophil killing. To evaluate the possible impact of NETs on parasite killing, luciferase-expressing WT, MIL-R and MA-R *L. (V.) p* parasites were exposed to netting neutrophils in presence or absence of DNAse. Four hours later, the frequency of viable luciferase expressing parasites was determined as previously described (28). The presence of DNAse did not change the relative number of WT parasites, suggesting no impact of NETs on parasite viability. In contrast, treatment of NETs with DNAse significantly increased the number of MA-R and even more so that of MIL-R *L.(V.) p* parasites, suggesting partial killing of the parasites by NETs (Figure 6D). Taken together, our data demonstrate that in both murine and human neutrophils, drug-resistant *L. (V.) p*. lines induce more mechanisms known to kill pathogens than the drug-susceptible parasites, yet they are better able to resist neutrophil killing even though partial killing by NETs was observed.

## Discussion

Anti-leishmanial drug-resistance is an increasing concern in Colombia, but also worldwide (45–47). In India miltefosine has replaced antimony as the first line of treatment against visceral leishmaniasis due to widespread evidence of antimony-resistance (48). However, the efficacy of miltefosine has also dropped considerably during the first 10 years following its introduction (49–51). It is crucial to consider that parasite drug-resistance is not equal to clinical treatment failure. Therapeutic response is also determined by host factors, such as previous infection, co-morbidities, nutritional status, pharmacokinetics etc. (52). Nevertheless, drug-resistance is a very important determinant of treatment failure, thus understanding how drug-resistance phenotype of *L. (V.) p*. impacts the anti-leishmanial immune response is relevant to the understanding of the potential role of neutrophils in therapeutic outcome.

Here, we have demonstrated for the first time that *L. (V.) p*. parasites having different drug-susceptibility phenotypes elicit distinct neutrophil effector functions, shown by a higher induction of ROS production, NET formation and the expression of surface markers characteristic of neutrophil activation. ROS production is a crucial microbicidal mechanism of neutrophils, but the impact of ROS on *Leishmania* survival is species and host dependent (53). Here, we showed that *L. (V.) p*. induces ROS production in murine and human neutrophils and that ROS production is increased when murine and human neutrophils are infected with the miltefosine-resistant *L (V.) p*. compared to the drug-susceptible parasites. In contrast, exposure of neutrophils to MA-R *L. (V.) p*. parasites had no significant impact on ROS production, suggesting that increased ROS production is not a general feature of parasites resistant to different drugs.

It has been reported that ROS production is critical for the induction of ROS-dependent NET formation (54). Activation and nuclear translocation of neutrophil elastase are crucial in ROS-dependent NET formation, reviewed in Papayannopoulos (55) and Brinkmann (56). However, there is also an NOX-independent pathway of NET formation that does not depend on ROS (57). *L. amazonensis* has been reported to induce ROS-independent and ROS-dependent NET formation. In our study, we show that MA-resistant and MIL-resistant *L. (V.) p*. induce increased in NET formation compared to that of susceptible strains, however only MIL-resistant *L. (V.) p*. induced an increased ROS production compared to that obtained from the drug-susceptible strain. These data suggests that the NET formation induced by MA-resistant *L. (V.) p*. may be partly ROS-independent.

The distinct activation profiles elicited by parasites having a different drug resistance phenotype are consistent with the absence of cross resistance i.e., Sb resistant parasites are not also resistant to miltefosine and vice-versa nor is there implication of a generic multi drug resistance mechanism. Resistance to different drugs is likely the outcome of the convergence of host cell and parasite responses to infection, their response to the drug, and drug effects on the host response. Our data implicate an association between the characteristics of the resistant and susceptible parasite population and neutrophil response. These characteristics could segregate with the susceptibility phenotype without being directly and causally involved in drug susceptibility. The neutrophil response elicited could enable the microbicidal effect of the drug, or drug resistance could provide a protective effect to neutrophil defense mechanisms as described for Sb resistance and lower nitric oxide susceptibility reported for some *Leishmania* spp. (58, 59).

Neutrophil effector functions contribute to microbial destruction in most infections, however, their role in leishmaniasis varies, depending on host factors and the *Leishmania* spp. involved (21, 53). We observed *L. (V.) p*. parasites associated with the filamentous structures of NETs, which substantiates parasite trapping by these structures. Most *Leishmania* spp. are trapped by NETs. Some, such as *L. mexicana* (28), *L. donovani* (60) *or L. infantum* (61) survive NET exposure, while others such as *L. amazonensis* (61–63) are killed by NETs, at least in their promastigote stage. The higher survival of drug-resistant parasites despite induction of greater ROS and NET response suggests that these *L. (V.) p*. lines resist better to neutrophil killing than *L. (V.) p*. susceptible strains. This is in line with studies performed on macrophages, reporting that MA as well as MIL-resistant *Leishmania donovani* strains were more resistant to ROS mediated killing due to increased intracellular thiol-levels (64–68). Neutrophil recruitment and increased neutrophil activation are known to play a key role in many inflammatory diseases (69, 70). Our results demonstrate that there is an increase in neutrophil activation upon stimulation by drug-resistant *L. (V.) p*. compared to drug-susceptible parasites. It is therefore conceivable that treatment failure may result at least in part due to the increased resistance of *L. (V.) p*. strains to neutrophil effector functions. An increase in parasite survival would then lead to an increase of inflammation and in turn would lead to more neutrophil recruitment and activation, and potentially constitute a self-sustaining circle.

In this study, we used laboratory-derived *L. (V.) p*. drug-resistant lines. Since there are differences in the drug-resistance mechanisms of parasites that develop drug-resistance *in vivo* compared to those experimentally selected for drug-resistance *in vitro* (7, 71) the assessment of the interaction of neutrophils and *L. (V.) p*. with different drug-susceptibility phenotypes that have been isolated from patients in a clinical setting should further elucidate of the role of the interaction of *L. (V.) p*. and neutrophils in anti-leishmanial responses. Also, the study of the *in vivo* immune response of neutrophils against those parasites would contribute to better understanding of the role the immune system plays in anti-leishmanial treatment outcome.

The concurrence of the present findings in mouse and human neutrophils strengthens the observations and provides a surrogate model to identify the mechanisms involved, in follow-up investigations based on these findings. Further understanding of the effect of drug-resistant parasites on neutrophil function, including the analysis ROS production and NET formation *ex vivo* (e.g., neutrophils from lesion biopsies or patient blood samples), should be further investigated to assess ehether this neutrophil activation could constitute a surrogate marker for treatment outcome.

## Author Contributions

IR and FT-C conceived the experiments. NS, OF, and MG contributed to the design and interpretation of the experiments and provided and authenticated the *L. (V.) p*. strains and lines of defined drug susceptibility phenotype. IR, OF, and BM-S performed the experiments and analyzed the data. IR wrote the manuscript, FT-C, OF, BM-S, MG, and NS contributed to and critically reviewed the manuscript.

### Conflict of Interest Statement

The authors declare that the research was conducted in the absence of any commercial or financial relationships that could be construed as a potential conflict of interest.
